# Assessing the detection of human papillomavirus late mRNA in liquid base cytology samples for risk stratification of cervical disease

**DOI:** 10.1002/jmv.23793

**Published:** 2013-10-19

**Authors:** George Chambers, David Millan, Kate Cuschieri, Heather A Cubie, Sheila V Graham

**Affiliations:** 1 MRC-University of Glasgow Centre for Virus Research Institute of Infection Immunity and Inflammation College of Medical, Veterinary and Life Sciences, University of GlasgowGlasgow, Scotland, UK; 2 Department of Pathology, Southern General HospitalGlasgow, UK; 3 Scottish HPV Reference Laboratory, Royal Infirmary of EdinburghEdinburgh, UK

**Keywords:** human papillomavirus, gene expression, cervical intraepithelial neoplasia, liquid based cytology, mRNA tests

## Abstract

Molecular human papillomavirus (HPV) testing is an important and developing tool for cervical disease management. However there is a requirement to develop new HPV tests that can differentiate between clinically significant and benign, clinically insignificant infection. Evidence would indicate that clinically significant infection is linked to an abortive HPV replication cycle. In particular the later stages of the replication cycle (i.e., production of late messenger (m) RNAs and proteins) appear compromised. Compared to current DNA-based tests which indicate only presence or absence of virus, detecting virus mRNAs by reverse transcriptase PCR (RT-PCR) may give a more refined insight into viral activity and by implication, clinical relevance. A novel quantitative (q)RT-PCR assay was developed for the detection of mRNAs produced late in the viral replication cycle. Initially this was validated on HPV-containing cell lines before being applied to a panel of 223 clinical cervical samples representing the cervical disease spectrum (normal to high grade). Samples were also tested by a commercial assay which detects expression of early HPV E6/E7 oncoprotein mRNAs. Late mRNAs were found in samples associated with no, low and high grade disease and did not risk-stratify HPV infection. The data reveal hidden complexities within the virus replication cycle and associated lesion development. This suggests that future mRNA tests for cervical disease may require quantitative detection of specific novel viral mRNAs. ***J. Med. Virol. 86:627–633, 2014*.** © 2013 Wiley Periodicals, Inc.

## INTRODUCTION

Persistent infection with “high risk” human papillomavirus (HR-HPV) genotypes is a necessary cause of cervical and other anogenital cancers [Walboomers et al., 1999; Munoz et al., 2003; Woodman et al., 2007; Bodily and Laimins, 2011]. Cytology-based cervical screening programs are only protective in part and lack sensitivity. HPV DNA testing has been introduced in several counties to improve sensitivity of cervical disease detection. Most HPV DNA tests used for cervical disease management are based on PCR detection of the L1 gene region. Although the tests are sensitive they cannot separate those infections that are clinically significant from those which are clinically irrelevant [Cuzick et al., 2006]. Thus more sophisticated tests are required to improve the specificity of HPV testing. Basic research can inform the development of such tests. Molecular studies of the HPV replication cycle have led to a number of key insights [Doorbar, 2005]. First, increased expression of HPV E6/E7 oncoproteins underpins cervical tumorigenesis. Second, productive infections where progeny virions are synthesized follow a full viral gene expression program (early and late events) and are rarely associated with malignancy. Third, persistent infections that can lead to malignancy are those where normal cell function is abrogated and where late events in the viral replication cycle are not supported or are supported only poorly.

Importantly, only active infections are clinically relevant. Their hallmark is production of viral messenger (m)RNAs and proteins. The low level of virus protein expression during infection and lack of good antibodies makes protein detection difficult. However, viral mRNAs encoding the proteins can be detected by reverse transcriptase (RT) PCR.

Tests to detect E6/E7 mRNA are available already. Increased expression of E6/E7 mRNA is hypothesized to mark clinically significant infections. Indeed, E6/E7 mRNA tests may show enhanced specificity in detecting clinically significant disease compared to DNA tests [Spathis et al., 2012; Cuzick et al., 2013]. However, E6/E7 mRNA detection itself is not entirely specific for clinically significant infection because E6/E7 mRNA expression seems to occur in both productive and persistent infection [Arbyn et al., 2013]. HPV capsid (late) proteins L1 and L2 expressed in the uppermost layers of the epithelium, are the ultimate markers of a productive infection, and are produced at reduced levels in persistent abortive infections [Middleton et al., 2003]. The capsid proteins are encoded by the viral late mRNAs that all contain L1 RNA sequences. With particular reference to HPV16, the hypothesis that late mRNA (L1) detection would identify productive infections less likely to be associated with significant underlying disease was tested.

## MATERIALS AND METHODS

### Clinical Sample Panel

The clinical panel constituted archived (stored at −80°C) cervical liquid based cytology (LBC) samples obtained from 223 women who had attended a colposcopy (all referral) clinic at the Simpsons Centre for Reproductive Health, Royal Infirmary of Edinburgh. Ethical approval for sample use was provided by Fife and Forth Valley Research Ethics Committee (reference number 07/S0501/92). Informed consent was obtained for sample collection and use. The panel selected for the study was composed of HPV-positive samples, representing the disease spectrum ranging from normal to cervical intraepithelial neoplasia grade 3. HPV status was confirmed by the Aptima HPV Assay (AHPV assay, GenProbe, San Diego, CA), a broad spectrum qualitative HPV E6/E7 mRNA test that detects 14 high risk (or putatively high risk) types in aggregate. All experiments were performed in compliance with relevant laws and institutional guidelines and in accordance with the ethical standards of the Declaration of Helsinki.

### Growth of Cell Lines

#### W12E (clone 20863)

Immortal cell line derived from a low grade cervical lesion [Stanley et al., 1989; Jeon et al., 1995] containing episomal HPV16 genomes. Epithelial cells were co-cultured in E-medium with mitomycin C-treated J2 3T3 fibroblast feeder cells at a ratio of 1:5, seeding keratinocytes at 2 × 10^5^ cells/100 mm dish [Jeon et al., 1995]. Cells were cultured for 10 days with 1.2 mM Ca^2+^ to allow epithelial differentiation and production of virus capsid proteins [Milligan et al., 2007].

#### W12G cells (clone 20861)

Immortal cell line derived from W12E containing mostly integrated HPV16 genomes [Jeon et al., 1995]. W12G cells were cultured exactly as W12E cells.

#### W12GPXY cells

Transformed invasive cell line containing integrated HPV16 genomes derived from W12G cells [Aasen et al., 2003].

#### HaCaT cells

Immortalized keratinocytes without HPV DNA [Boukamp et al., 1988].

HaCaT and W12GPXY cells were grown in DMEM, 10% foetal bovine serum, 2 mM glutamine without feeder cells.

### RNA Extraction and RT-PCR Detection of HPV mRNAs

Prior to RNA extraction fibroblast feeder cells were removed from W12E and W12G cells by trypsinization. RNA from all cells was prepared using Trizol (Life Technologies, Carlsbad, CA) according to the manufacturer's protocol and DNA was removed by TURBODNase (Life Technologies) treatment according to the manufacturer's protocol. SuperScript III First-Strand Synthesis System for RT-PCR (Life Technologies) was used for cDNA synthesis. RT-negative controls contained RNA and all reagents except reverse transcriptase. Semi-quantitative PCR was used to simultaneously amplify cellular GAPDH and HPV L1 cDNA. Primers used are shown in Table1. Products were resolved on 6% acrylamide gels and visualized by staining with ethidium bromide.

**Table IA:** Primer Sequences for RT-PCR

Primer name	HPV genome position (nt)	Sequence 5′–3′
16L1-F	7003–7024	CTGCAGACCTAGATCAGTTTCC
16L1-R	7312–7289	GCATGACACAATAGTTACACAAGC
GAPDH-F	N/A	AGGAAATGAGCTTGACAAAG
GAPDH-R	N/A	ACCACAGTCCATGCCATCAC

F, forward primer; R, reverse primer.

### DNA Genotyping

Automated DNA extraction was performed on LBC samples using a Qiagen MDX machine in conjunction with the QIAamp Media MDX Kit (Qiagen, Manchester, UK). In order to characterize which samples harbored HPV16, PCR and genotyping of amplicons was performed using a luminex-based assay (Multmetrix HPV Genotyping Kit from Progen, Heidelberg, Germany) which can delineate the presence of 24 low and high risk HPVs including type 16 [Schmitt et al., 2006].

### Development of a qRT-PCR Assay for HPV L1

MIQE précis guidelines were followed for design and implementation of the qRT-PCR assay [Bustin et al., 2010].

#### RNA extraction—clinical samples

Cells from 4 ml LBC cell suspensions in PreservCyt collection medium (Cytyc Corporation, Marlborough, MA) were pelleted by centrifugation in a Beckman GPR bench top centrifuge at 1,500*g* for 10 min. The supernatant was decanted and the cell pellet washed with sterile PBS pre-treated with 0.1% v/v DEPC (Sigma, Poole, UK). RNA extraction was optimized on aliquots of the clinical samples. Three RNA extraction kits: RNeasy (Qiagen) and RNeasy FFPE (Qiagen) and MasterPure (Life Technologies), were evaluated. RNA was quantified and purity assessed by measuring the ratio of absorbance at 260 and 280 nm using a Nanodrop ND-1000 spectrophotometer. Integrity of selected RNAs was assessed using an Agilent 2100 Bioanalyser. MasterPure gave best yields and purity of RNA.

#### cDNA synthesis

cDNA synthesis was performed on DNase-treated extracted nucleic acid using a Stratagene AffinityScript qPCR cDNA synthesis kit (Agilent Technologies, Santa Clara, CA) in accordance with the manufacturer's instructions. RT-negative controls containing RNA and all reagents except reverse transcriptase were prepared for all samples.

#### Primer/probe selection

The specificity of eight different probe/primer sets within the L1 gene region was validated in silico (BLAST). Optimal annealing temperature was assessed by gradient PCR and primer and Mg^2+^ concentrations were also optimized using cDNA prepared from RNA isolated from W12E cells (active HPV16 infection). The primers were evaluated in qRT-PCR using serial dilutions of W12E cell RNA. The optimal probe-primer set was L1-1 (Table2) with a threshold cycle (Ct) value compatible with the control β-actin and GAPDH probe/primer sets (98% efficiency). For analysis of the LBC samples qRT-PCR primers and FAM/TAMRA dual labeled probes were supplied by Eurogentec (Southhampton, UK). qRT-PCR Mastermix was Stratagene Brilliant qPCR Mastermix (Agilent Technologies), reference dye was ROX supplied with the Mastermix. For each primer/probe set the reaction contained 1× Mastermix, 900 nM forward primer, 900 nM reverse primer 100 nM probe, 300 nM ROX in a final volume of 23 µl. Reactions were carried out in Thermofast 96 ml × 0.2 ml well non-skirted PCR plates (Thermo Fisher, Loughborough, UK). Addition of 2 µl template to each well was used to start the reactions. Quantity of template was 0 (non-template control) or 10 ng cDNA or 10 ng cDNA reaction without reverse transcriptase added to test for presence of residual DNA. All samples were assayed in triplicate. A calibrator consisting of 10 ng of cDNA prepared from RNA extracted from 3.14 × 10^3^ W12E cells mixed with 2.6 × 10^6^ HaCaT cells was used on every plate. qRT-PCR results were plotted as Ct value versus log dilution factor. qRT-PCR was carried out on an Applied Biosystems (Life Technologies, Carlsbad, CA) 7500 real time PCR machine with the following thermocycling conditions: 50°C for 2 min; 95°C for 10 min; then 40 cycles of 95°C for 15 sec, 60°C for 1 min; well volume 25 µl. Applied Biosystems software was used for data analysis.

**Table IB:** Primer and Probe Sequences for qRT-PCR

Primer name	HPV genome position (nt)	Sequence 5′–3′
16L1-1-F	6576–6593	CAACGAGCACAGGGCCAC
16L1-1-R	6661–6688	GAAGTAGATATGGCAGCAC
16L1-1 Probe	6615–6650	CCAACTATTTGTTACTGTTGTTGATACTACACGCAG
Actin-F	N/A	GGGATGTTTGCTCCAACCAA
Actin-R	N/A	GCGCTTTTGACTCAAGGATTTAA
Actin probe	N/A	CGGTCGCCTTCACCGTTCCAGTT
GAPDH-F	N/A	GAAGGTGAAGGTCGGAGT
GAPDH-R	N/A	GAAGATGGTGATGGGATTTC
GAPDH probe	N/A	CAAGCTTCCCGTTCTCAGCC

F, forward primer; R, reverse primer. Probes were FAM/TAMRA labeled.

### E6/E7 mRNA Type Specific Testing

Samples that were shown to harbor HPV16 infection were also tested with the PreTect HPV Proofer (Norchip A, Klokkarstua, Norway). This is a NASBA-based assay which incorporates the qualitative detection of full-length E6/E7 mRNA for 5 HR-HPV types using molecular beacons (16, 18, 31, 33, and 45).

## RESULTS

### Evaluation of LBC mRNA and q-RT-PCR for Late mRNA Detection

Eight Taqman primer and probe sets covering different regions of the L1 ORF were designed and evaluated using cDNA prepared from W12E cells, which are cervical intraepithelial neoplasia grade 1-derived cells with the HPV16 genome in the episomal form. Standard curves were plotted and efficiencies for all of these sets were calculated and compared to those for concurrent standard curves prepared from existing primer and probe sets for β-actin and GAPDH, two housekeeping genes. Three of the L1 primer/probe sets were found to have compatible efficiency with the control primer/probe sets and of these, the one with the lowest Ct value at the log relative dilution factor of 0 (neat sample) was chosen as the set to use to assay the LBC samples. The amplicon (Table2) was located in the last third of the L1 open reading frame which is present in all late mRNAs. Since the L1 and β-actin and GAPDH primer/probe sets had comparable efficiencies, the ddCt method of relative quantification was employed.

**Figure 1:**
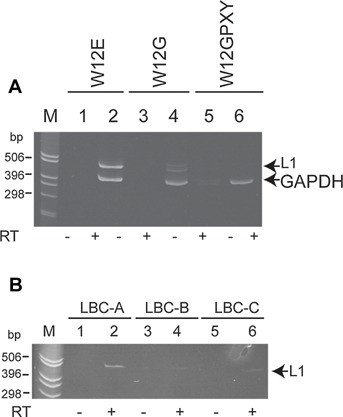
HPV16 late mRNA can be detected in cervical cell lines that support the complete viral replication cycle and in mRNA prepared from archived LBC samples. **A**: RT-PCR amplification (35 cycles) of HPV16 late mRNA from W12E (episomal HPV16 genome), W12G (mainly integrated HPV16 genomes) and W12GPXY (fully integrated HPV16 genomes) cells using primer set 16L1-F/R (Table1). Detection of GAPDH cDNA was used as an internal control. **B**: RT-PCR amplification (35 cycles) of HPV16 late mRNA from three LBC samples. LBC samples are labeled A, B, and C to distinguish them from those used in Figure 2 (LBCs 1 and 2). M, marker track. (−) Amplification in the absence of reverse transcriptase. (+) Amplification in the presence of reverse transcriptase.

Detection of HPV16 late mRNA using the primer set in Table1 was tested in W12 cells as an in vitro model of HPV16 infection and in a small preliminary clinical panel of 8 LBC samples. Late (L1) mRNA was detected by semi-quantitative RT-PCR in W12E cells (Fig.1A, track 2). These cells were established from a low grade cervical lesion, contain ∼100 HPV16 episomal genomes and are capable of supporting the complete viral replication cycle [Stanley et al., 1989]. W12G and W12GPXY cells are models of persistent, abortive HPV infection. Only a faint band representing late mRNA was detected in W12G cells (Fig. 1A, track 4), where most HPV16 genomes are integrated into the host genome [Jeon et al., 1995] and no late mRNA was detected in W12GPXY cells (Fig. 1A, track 6) that contain only integrated HPV genomes and are fully transformed and invasive [Aasen et al., 2003]. GAPDH was used as an internal loading control in this assay. Late mRNAs were also detected in three (LBC-A, B, and C) out of eight HPV16-positive archived LBC samples. A representative RT-PCR analysis is shown in Figure 1B. GAPDH control was not detected in this experiment.

### RNA Quality in LBC Samples

**Figure 2:**
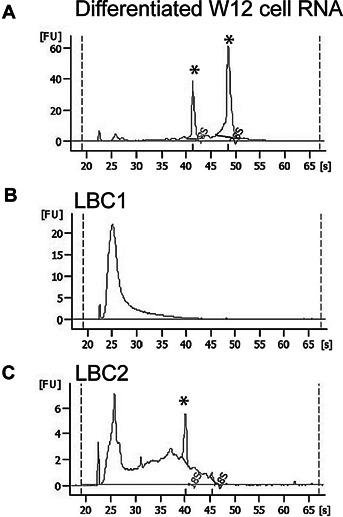
Quality of RNA isolated from archival LBC samples. RNA was analyzed on an Agilent 2100 bioanalyzer. **A**: RNA isolated from differentiated W12E cells. Peaks at 18S and 28S corresponding to rRNAs are indicated (asterisks). **B**: Poor quality RNA isolated from an LBC sample. **C**: RNA from a different LBC sample showed a wider range of sizes with a detectable 18S ribosomal RNA peak (asterisk).

Overall, RNA quality from the archived LBC samples was extremely variable. Figure 2 shows a comparison of bioanalyzer profiles of freshly prepared W12E RNA (Fig. 2A) and RNA prepared from two archived LBC samples (Fig. 2B,C). Ribosomal RNA peaks were predominant in the W12E RNA sample but absent in one of the clinical samples (LBC1) and partially degraded in the other (LBC2). Despite this, qRT-PCR was possible for all LBC samples with both the control β-actin and GAPDH probe/primer sets. Table3 shows the average value and range of values for detection of the controls in qRT-PCR of cDNAs synthesized from W12E cell RNA and from LBC RNAs. The average Ct values were greater for LBC samples as was the range of values as expected due to the variable quality of these RNAs.

**Table II:** Average and Range Ct Values for the Control β-Actin and GAPDH Amplifications in W12 Cells and in LBC Samples

Sample type	GAPDH		β-Actin	
	Average Ct value	Range of Ct values	Average Ct value	Range of Ct values
W12E cells	20.52	19.20–22.12	20.10	19.43–21.78
LBC samples	23.48	19.54–28.32	22.95	19.16–27.16

### Characterization of the Clinical Panel

HPV DNA typing using the luminex assay indicated HPV16-positivity in 98/223 archived LBC samples: 43 appearances with another type (or types) and as a mono-infection in 55 samples. As the late mRNA assay was designed to be specific for HPV16, further analysis was confined to the 98 HPV16-positive samples. Complete clinical data was available for 88 of the 98 samples: 45 were associated with no significant disease and comprised normal histology, normal colposcopy/inflammation only (no biopsy indicated) or cervical intraepithelial neoplasia grade 1. A total of 43 cases were associated with histologically confirmed high-grade disease (cervical intraepithelial neoplasia grade 2+).

### Relation of Late mRNA Positivity to Clinical Outcomes and E6/E7 mRNA Detection

HPV16 late mRNA qRT-PCR was performed blinded to the underlying pathology. Of the 98 HPV16 DNA positive samples, 82 were negative for the L1 qRT-PCR and 16 were positive. Of the positives, 9/16 (56.2%) were associated with high-grade disease and 7/16 (43.7%) were associated with no or low grade disease (Table4). In the case of multiple HPV infections it is challenging to attribute the HPV type responsible for any abnormality, we therefore stratified qRT-PCR status according to disease and mono- or multiple-infection (associated with HPV16). A total 9 of the 16 L1 qRT-PCR positive samples were associated with HPV16 mono-infection, of which 6 were associated with high grade disease and 3 were associated with no disease (Table4). Therefore L1 mRNA detection did not, in itself risk-stratify HPV infections according to cross sectional disease status.

**Table III:** Late mRNA Expression in 98 HPV16 Positive Samples and Association With Clinical Outcomes*

Grade	Number of cases overall	Late mRNA positive overall	Number of cases associated with mono HPV16 infection	Late mRNA positive
Normal	36	7	19	3
CIN1	9	0	3	0
CIN2	20	6	10	3
CIN3	21	3	16	3
High grade undefined	2		1	0
Incomplete record/lost to follow-up	10		6	
Total	98	16	55	9

* All samples were collected from referral colposcopy clinics where biopsies were taken as routinely indicated. All samples had previously tested positive using a broad spectrum E6/E7 mRNA test.

Due to the variable quality of the RNA extracted from the archived LBC samples only 44 of the 98 HPV16 positive samples were evaluable by the E6/E7 Proofer assay (as evidence by lack of amplification of the internal control). The lack of detection of E6/E7 in all of the samples likely reflects the suboptimal quality of RNA in archived LBC samples and the fact that the U1A housekeeping amplicon is a low abundance housekeeping gene [Molden et al., 2007]. Of this subset 36 had available disease outcomes incorporating a total of 16 cervical intraepithelial neoplasia grade 2+ The proofer assay tested positive in 10 of these 16 cases (62.5%) and 12 of the 20 cases where no or low grade disease was detected (60%). Only 4/44 of the samples that were evaluable by proofer tested positive for the L1 qRT-PCR; none of which were associated with high-grade disease. Of the 4 L1 qRT-PCR positives, 3 were also positive by the proofer.

## DISCUSSION

The presence of HPV late proteins in the upper layers of infected epithelia has been proposed as a negative prognostic marker of disease severity [Middleton et al., 2003]. Early in situ hybridization studies showed HR-HPV late RNAs were present in cells of the upper epithelial layers in low grade cervical lesions [Crum et al., 1988; Beyer-Finkler et al., 1990; Stoler et al., 1992] and absent in high grade lesions [Stoler et al., 1992]. Late events in the HPV replication cycle, including late mRNA production, are tightly linked to terminal epithelial differentiation, so reduced differentiation capacity—a hallmark of high grade lesions—could lead to abortive infection and diminished late mRNA expression [Middleton et al., 2003]. Viral late mRNA was able to be detected by a technically validated L1 qRT-PCR in LBC samples representing different grades of disease but the data show that detection of late mRNA did not risk-stratify samples into a “lower” risk group according to underling disease status. There are caveats to the study/interpretation. First, archived samples were used where the quality of recoverable RNA is highly variable and significantly less than that of freshly collected samples [Cuschieri et al., 2005]. Further validation using freshly collected LBC samples would be beneficial. Second, multiple infections confound type specific-attribution for underlying pathology. However, when analysis was restricted to samples associated with a mono-infection of HPV16, surprisingly, L1 mRNA detection was proportionally more abundant in samples associated with high grade disease compared to low-grade, or no disease. Third the clinical correlations were entirely cross-sectional; it is feasible that longitudinal assessment could have revealed differential outcomes associated with gene expression.

In this study, the performance of the L1 mRNA PCR was not assessed using standard clinical performance measures of sensitivity, specificity PPV and NPV. While such measures (and the studies of prospective populations from which they are derived) are essential for biomarker validation it is also essential they are informed, a priori, with pilot, proof-of-principle studies that demonstrate a correlation between the biomarker under investigation and disease. This study constituted such a pilot and provided valuable insight into the limitations of L1 mRNA PCR.

E6/E7 oncogene mRNA expression is expected to increase with lesion severity while expression of viral late mRNAs should be greatest in an infectious situation, that is, low grade disease [Issacson-Wechsler et al., 2012]. This study indicates that the pattern of clinically significant viral gene expression is more complex than this and that E6/E7 mRNA and L1 mRNA can be detected both in infections associated with no disease and in high grade disease, limiting the ability of these approaches for comprehensive risk-stratification in their current form [Schmitt et al., 2011; Arbyn et al., 2013]. The data highlight lack of detailed knowledge of HPV gene expression in the cervical epithelium during disease progression and prior to cancer formation. In addition, lesion pathology may be complex [Quint et al., 2012] with multiple simultaneous HPV infections, various viral loads and different grades of disease within a single cervical epithelium. Viral gene expression is also altered depending on the episomal (the infectious form of the virus genome) or integrated (found more frequently in high grade lesions) status of the viral genome and each cervical lesion may exhibit mosaicism in this respect. On top of this, epigenetic events impacting the host or viral genomes may also impinge on viral gene expression at the single cell level, creating an additional level of complexity [Szalmás and Kónya, 2009]. The relative stabilities of key viral mRNAs such as E6/E7 and late mRNAs may also change during cervical disease progression and confound detection strategies [Graham, 2010]. The low prevalence of late mRNAs supports evidence from several studies on a number of animal and human papillomaviruses that showed late mRNAs are unstable due to the activity of an mRNA decay element in the late 3′ untranslated region [Graham, 2008] and recent deep sequencing data demonstrating very low levels of late mRNAs in cell models of HPV16-positive low grade cervical lesions [Klymenko and Graham, manuscript in preparation].

## CONCLUSIONS

A late mRNA assay for detection of HPV16 infection applicable to liquid based cytology samples was developed. Although the assay did not differentiate clinically insignificant infection, this is the first molecular study to interrogate its potential in a well characterized context where HPV status and clinical data were available. Selective primer/probe sets that can distinguish individual viral mRNAs and quantify expression of these compared to bulk viral mRNA expression may prove more useful as biomarkers of disease [Schmitt et al., 2010]. It is clear that in future it would be valuable to interrogate the amplification of viral oncogene mRNAs and late mRNAs in more contemporary or freshly acquired prospective LBC samples.

## Abbreviations

HPV: human papillomavirus
HR-HPV: high risk-HPV
LBC: liquid based cytology
RT-PCR: reverse transcriptase polymerase chain reaction
AHPV: Aptima HPV Assay

## Supporting Information

Fig S1 Standard curves of amplification of HPV16 late mRNA using primer set 1 (**A**) and β-actin (**B**) using W12E cDNA. Very similar data were obtained with GAPDH. **C**: A relative efficiency plot of amplification of β-actin versus L1 in the same HPV16-positive cells is shown. **D**: Calibration curve of detection of HPV16 late mRNA in log dilution populations of W12E cells in a background of HPV-negative HaCaT keratinocytes. The cell population containing 3.14 × 10^3^ W12E cells in 2.6 × 10^6^ HaCaT cells was chosen as the calibrator for the qRT-PCR assays it was at this point that the 1000× normalized L1 value leveled out, representing the sensitivity limit of the assay.
